# Analysis of clinical cure outcome, macrophages number, cytokines levels and expression of annexin-A1 in the cutaneous infection in patients with *Leishmania braziliensis*

**DOI:** 10.1590/0037-8682-0036-2024

**Published:** 2024-07-29

**Authors:** Joselina Maria da Silva, Helen Aguiar Lemes da Silva, Ana Lucia Carneiro Sarmento, Marcia Hueb, Amílcar Sabino Damazo

**Affiliations:** 1 Universidade Federal de Mato Grosso, Faculdade de Medicina, Programa de Pós-graduação em Ciências da Saúde, Cuiabá, MT, Brasil.; 2 Universidade de Brasília, Faculdade de Medicina, Brasília, DF, Brasil.; 3 Universidade Federal de Mato Grosso, Faculdade de Medicina, Departamento de Clínica Médica, Cuiabá, MT, Brasil.

**Keywords:** Leishmania braziliensis, Cutaneous leishmaniasis, Cytokines, Annexin-A1, Histopathology

## Abstract

**Background:**

*Leishmania braziliensis*, a protozoan prevalent in Brazil, is the known causative agent of cutaneous leishmaniasis (CL). The activation of M1 macrophages is a pivotal factor in the host's ability to eliminate the parasite, whereas M2 macrophages may facilitate parasite proliferation. This study analyzed the clinical outcomes of CL and the patients' immunological profiles, focusing on the prevalence of M1 and M2 macrophages, cytokine production, and annexin-A1 (ANXA1) expression in the lesion.

**Methods:**

Data were obtained by polymerase chain reaction (PCR) and histopathological, immunofluorescence, and cytokine analyses.

**Results:**

Patients with exudative and cellular reaction-type (ECR)-type lesions that healed within 90 days showed a significant increase in M1. Conversely, patients with ECR and exudative and granulomatous reaction (EGR)types, who healed within 180 days, showed an elevated number of M2. Cytokines interferon (IFN)-γ and tumor necrosis factor (TNF)-α were higher in ECR lesions that resolved within 90 days (P<0.05). In contrast, IL-9 and IL-10 levels significantly increased in both ECR and EGR lesions that healed after 180 days (P<0.001). The production of IL-21, IL-23 and TGF-β was increased in patients with ECR or EGR lesions that healed after 180 days (P<0.05). The expression of ANXA1 was higher in M2 within ECR-type lesions in patients who healed after 180 days (P<0.05).

**Conclusions:**

These findings suggest that the infectious microenvironment induced by L. braziliensis affects the differentiation of M1 and M2 macrophages, cytokine release, and ANXA1 expression, thereby influencing the healing capacity of patients. Therefore, histopathological and immunological investigations may improve the selection of CL therapy.

## INTRODUCTION

Cutaneous leishmaniasis (CL) is a clinical condition caused by the parasites from the genus *Leishmania*. In Brazil, CL is characterized by ulcerated lesions that typically exhibit oval or rounded shapes. *Leishmania braziliensis* is one of the most common parasites in Brazil. These lesions are characterized by a granulomatous foundation and possess well-defined elevated edges^1^ ^,^ ^2^. Notably, CL lesions primarily affect exposed body areas, which can result in permanent scars, disfigurement, stigma, and in some cases, disability^3^.

Classified as a neglected tropical disease, CL is estimated to cause 222,000 new cases worldwide by 2022^3^. In Brazil, 12,878 new cases of CL were documented this year, of which 1,162 were reported in the state of Mato Grosso^4^.

Several factors can affect CL treatment, such as diversity among *Leishmania* species and the complexity of the host's immune system of the host^2^ ^,^ ^5^ ^,^ ^6^. Therefore, a comprehensive understanding of the host immune response to *Leishmania* is essential for the advancement of drug discovery and the development of novel therapeutic approaches.

Macrophages play a role in the regulation of adaptive immunity and are crucial components of the innate immune system^2^. These cells respond to a wide range of environmental signals by producing molecules that modulate the host's response to the parasite, including defense against infectious processes and wound healing. Macrophages can undergo differentiation depending on the infecting *Leishmania* species and infectious microenvironment^7^ ^,^ ^8^.

Macrophage M1 activation, leads to a classical profile described by the secretion of pro-inflammatory molecules, for example, as interferon (IFN)-γ, tumor necrosis factor (TNF)-α, interleukin (IL)-1, IL-6, and IL-23, and the production of reactive oxygen species (ROS) and nitrogen radicals. These cells integrate the Th1 response to eliminate microorganisms^9^ ^-^ ^12^.

In contrast, macrophage M2 activation, results in an alternative profile that is characterized by the upregulation of scavenger, mannose, and galactose receptors, as well as the expression of antagonists of the IL-1 receptor, and negatively regulates IL-1β and other pro-inflammatory cytokines. This condition allows an increases the number of parasites and exacerbates the disease^13^ ^-^ ^15^. M2 macrophages play a pivotal role in protecting the host from excessive inflammation and promoting tissue repair and wound healing^6^.

Several studies identified M1 and M2 macrophages in the skin of patients with *L. braziliensis*. These studies observed no differences in the number of M1 and M2 cutaneous lesion^7^ ^,^ ^16^. These studies also showed that lesions caused by *L. braziliensis* were primarily composed of T lymphocytes, plasma cells, and macrophages, and exhibited a low level of parasitism. The predominant cytokine activation is the Th1 response, which is likely driven by parasite antigens^7^ ^,^ ^15^. Additionally, some studies^16^ ^,^ ^17^ have characterized the presence of annexin-A1 (ANXA1), an anti-inflammatory protein that functions as a critical regulatory molecule in the immune response. ANXA1 plays an important role in mediating both the activation and migration of leukocytes, which are essential for orchestrating the immune system response to infection and inflammation^18^ ^,^ ^19^. The presence of ANXA1 in *Leishmania braziliensis* infection suggests its potential involvement in modulating the host immune response, possibly favoring parasite survival^16^ ^,^ ^17^. 

This study aimed to investigate the clinical outcomes of a cure for CL and immunological molecules during infection, as determined by the identification of M1 and M2 macrophages at inflammatory lesions, local cytokine levels, and ANXA1 expression.

## METHODS

### ● Patients

Patients diagnosed with CL (N = 120) were invited to participate in the study. They visited the Leishmaniasis Outpatient Clinic of Júlio Müller University Hospital (HUJM) in Cuiabá, Brazil. This outpatient clinic was selected because it serves as a state reference for the diagnosis and treatment of leishmaniasis. The eligible participants for this study were patients with CL who had not initiated CL treatment. Patients with immunosuppressive conditions or infectious or chronic degenerative diseases were excluded. In addition, patients should have suggestive histopathological characteristics of CL and the identification of *Leishmania braziliensis* by molecular characterization.

Fifty patients were included in this study. Their average age was 44 years (18-56 years). 

Information regarding residence, age, sex, origin, symptom onset, type of drug used, and end date of treatment was collected. All the patients underwent a general physical examination. The medical team at HUJM assessed the general health condition of the patients and lesion characteristics, including edges, size, shape, and location.

All patients were informed about the procedures and aims of the study, and their freedom to participate. Patients who agreed to participate signed the Informed Consent Form approved by the Research Ethics Committee of HUJM (CEEA no.51430915.0.0000.55.41). This study posed no additional risk, as the collected material (biopsy) was the same as that used in routine laboratory tests.

All patients received intravenous glucantime (meglumine antimoniate) at a concentration of 20 mg/kg/day for 20 days^1^. Patients cured for up to 90 d were included in this study. If the lesion persisted, the patient received a second dose of meglumine antimoniate^1^. Patients cured for up to 180 d were included in this study.

### ● Laboratory exams

For a positive diagnosis of CL, a biopsy was performed for histopathology, and a cervical brush was used at the edge of the lesion for polymerase chain reaction (PCR).

The skin was aseptically cleaned with an iodine solution and subsequently with a 0.9% saline solution. The area was injected with 2% lidocaine, and the biopsy specimen was collected using a 4 mm punch.

### ● Collection of cervical brush samples, DNA extraction, and *Leishmania* species identification with PCR-HSP70C

After collection using cervical brushes, the sample was placed in a tube containing phosphate-buffered saline (PBS).

Commercial kits for DNA extraction (Invitrogen) were used according to the manufacturer's instructions. The extracted DNA was quantified using a Nanodrop.

The method used for *Leishmania* species identification was PCR-HSP70C^17^, using forward primer: 5`GGACGAGATCGAGCGCATGGT´3 and reverse primer: 5`TCCTTCGACGCCTCCTGGTTG´3. The PCR-HSP70C samples were electrophoresed on a 2% agarose gel.

For all samples with positive visualization, *Leishmania* species were identified using PCR-restriction fragment length polymorphism (RFLP). Samples were incubated for 12 h at 37°C with the enzymes HaeIII and BstUI^17^. The resulting band was compared with three references for *Leishmania* species (*Leishmania* Collection of Oswaldo Cruz Institute, CLIOC), following WHO standards, and a negative control (no DNA): *Leishmania guyanensis* (MHOM/BR/1975/M4147/IOC/L), *Leishmania braziliensis* (MHOM/BR/1975/M2903/IOC/L566); *Leishmania amazonensis* (IFLA/BR/1967/PH8/IOC/L575).

### ● Histopathological Analysis

Biopsy was performed in the histology laboratory at the Faculty of Medicine of the Federal University of Mato Grosso (UFMT). Samples were fixed in 4% paraformaldehyde in PBS solution, dehydrated in increasing concentrations of ethanol, clarified in xylene, included in paraffin, sectioned at 3 µm with the HIRAX M60 microtome (Carl Zeiss; Germany), placed on glass slides and, stained with hematoxylin-eosin with a differentiator for histopathological analysis. Histopathological classification of CL lesions was performed according to the method described by de Magalhães and colleagues^20^: exudative and cellular reaction (ECR), exudative and granulomatous reaction (EGR), exudative and necrotic reaction (ENR), exudative and necrotic-granulomatous reaction (ENGR), and exudative and tuberculoid reaction (ETR).

### ● Cytokine analysis

Biopsy samples were collected and immediatelly frozen in liquid nitrogen, macerated using a ceramic pestle, and diluted in PBS for the quantification of IFN-γ, TNF-α, IL-1β, IL-6, IL-9, IL-10, TGF-β, IL-17, IL-21, and IL-23. Milliplex® kit (BD, New York, USA) and a dual-laser flow-based detector MAGPIX (Luminex® XMAP Technology, Texas, USA) were used for cytokine detection.

### ● ANXA1 endogenous protein expression and cellular markers identification by immunofluorescence

ANXA1 and macrophages were detected as previously described^18^. To detect ANXA1, antibody rabbit anti-ANXA1 (Invitrogen, USA) was used, diluted with PBS/bovine serum albumin (BSA) 1% (1:200); for macrophages, mouse anti-CD163 (Cell Marque, USA; 1:200); for M1 macrophages, rat anti-MHCII (Santa Cruz Biotechnology Inc., USA; 1:100); and for M2 macrophages, rat anti-CD206 (R&D Systems; 1:50). The secondary antibodies used were goat anti-rabbit IgG conjugated with ALEXAFLUOR 488, goat anti-mouse IgG conjugated with ALEXAFLUOR 555, and goat anti-rat IgG conjugated with ALEXAFLUOR 647 (Invitrogen, USA). The nuclear stainer was the DAPI (4',6-diamidino-2-phenylindole) (Invitrogen™, USA). The cells were analyzed using an AxioScope A1 microscope and Axiovision Software (Carl Zeiss, GR). Various points in the macrophage cytoplasm were evaluated, and ANXA1 expression was measured in arbitrary units (a. u.) (0-255)^18^.

### ● Statistical analysis

For statistical analyses, the data were written as mean ± standard deviation (SD). Macrophage number, cytokine release, and ANXA1 expression were analyzed in relation to the histopathological type and clinical cure outcome using one-way ANOVA with Tukey’s post-hoc test. Statistically significant results were obtained with P values <0.05.

## RESULTS

### ● Clinical and histopathological data

**FIGURE 1:**
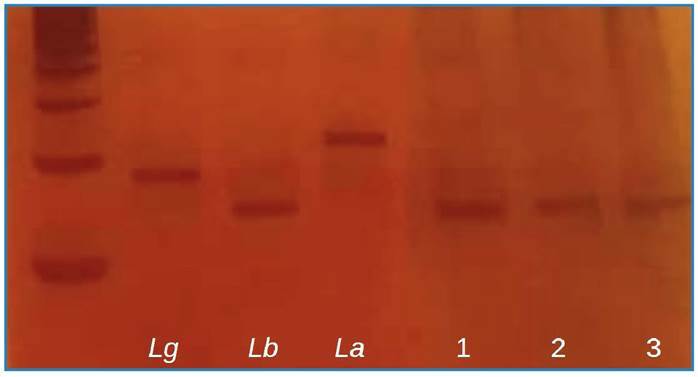
Identification of the *Leishmania* species by PCR-RFLP. Hae III enzyme digestion. Standard *Leishmania* species. ***Lg: Leishmania guyanensis Lb: Leishmania braziliensis; La: Leishmania amazonensis*** ; patients samples (1-3).

All patients tested positive for *L. braziliensis* infection (Figure 1). Regarding gender, 40 were male (80%) and 10 were female (20%). Regarding skin color, 40% of the patients were white, 30% were brown, and 30% were black. Most patients (34 %) presented with ulcerated infiltrated lesions with elevated edges and granulomatous bases. Most patients had lesions in the lower limbs (50%) (Table 1). In the histopathological analysis, the patients were classified by lesion type and evaluated for cure time after treatment with meglumine antimoniate (Table 1). Most patients with ECR and EGR lesions were cured after 180 days of meglumine antimoniate treatment, whereas only 50% of patients with ENR were cured after 180 days.

**TABLE 1:** Epidemiological, clinical, and histopathological data of cutaneous leishmaniasis.

Variables		Quantity	Percentage (%)
**Sex**	Male	40	80.0
	Female	10	20.0
**Skin colour**	Black	15	30.0
	White	20	40.0
	Brown	15	30.0
**Description of the injury**	Ulcerated with raised edges, granular bottom with exudate	5	10.0
	Ulcerated with raised edges, granular bottom	2	4.0
	Ulcerated and infiltrated	15	30.0
	Ulcerated	2	4.0
	Granulomatous	4	8.0
	Ulcerated, infiltrated, raised edge, granulomatous background	17	34.0
	Infiltrated	4	8.0
	Granulomatous and infiltrated	1	2.0
**Injury site**	Face	1	2.0
	Torso	6	12.0
	Upper limb	19	38.0
	Lower limb	25	50.0
**ECR**	cure up to 90 days	16	45.7
	cure up to 180 days	19	54.3
**EGR**	cure up to 90 days	3	33.3
	cure up to 180 days	6	66.7
**ENR**	cure up to 90 days	3	50.0
	cure up to 180 days	3	50.0

Consider the time of lesion previous to the treatment, the patients with ECR lesion autodeclare 2.8 ± 1.5 month, the paients with EGR 7.4 ± 6.0 month and the patients with ENR 3.5 ± 2.4 month.

### ● Analysis of macrophage subtype profile and cure of cutaneous leishmaniasis patients

Immunophenotyping of M1 and M2 cells was performed by immunofluorescence staining. Analysis of the number of macrophages in the lesion types and cure profiles revealed that patients with ECR lesions who were cured within 90 days had more M1 macrophages (P<0.05) than those cured within 180 days. Patients with ECR and EGR lesions who were cured within 180 days (P<0.001) had more M2 macrophages. Patients with ENR lesions had similar numbers of M1 and M2 macrophages regardless of whether they were cured for 90 or 180 days (Table 2).

**TABLE 2:** Healing time and number of M1 and M2 macrophages at lesions with cutaneous leishmaniasis.

Type of injury	Cure time	Macrophage M1	Macrophage M2
ECR	cure up to 90 days	35.5 ± 12.7 *	22.0 ± 6.3
	cure up to 180 days	23.5 ± 9.5	45.0 ± 11.1 ***
EGR	cure up to 90 days	31.5± 6.3	24.6 ± 6.3
	cure up to 180 days	30.5± 6.3	38.0 ± 6.3 ***
ENR	cure up to 90 days	35.5 ± 6.3	31.5 ± 6.3
	cure up to 180 days	38.5 ± 9.5	32.3 ± 12.7

Data was mean ± SD and was compared by One Way ANOVA, with Tukey post-test. * P<0.05 for 90 days versus 180 days; *** P<0.001 for 90 days versus 180 days.

### ● Analysis of cytokines and ANXA1 protein in cutaneous leishmaniasis patients

**FIGURE 2:**
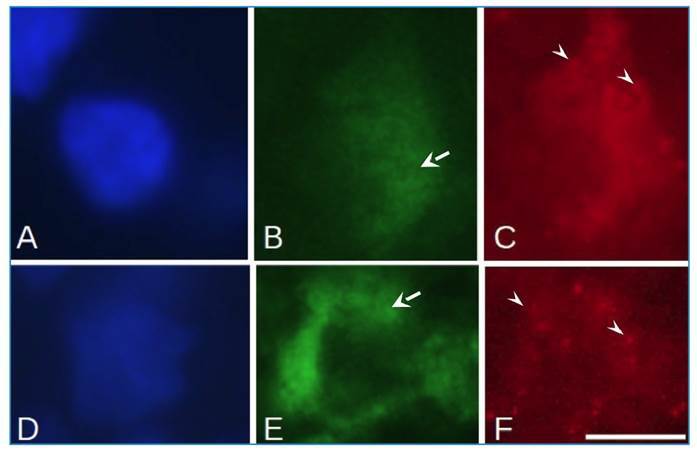
Immunofluorescence analysis of macrophages M1 and M2. **(A, B, and C)** Macrophage M1 in the patient's skin with leishmaniasis identified by MHC II stain (arrowhead) exhibited ANXA1 expression (arrow). **(D, E, and F)** Macrophage M2 identified by CD206 stain (arrowhead) also exhibited ANXA1 expression (arrow). **(A and D)** DAPI, **(B and E)** ANXA1 stain, **(C)** MHC II stain, and **(F)** CD206 stain. Bar = 5 μm.

The levels of cytokines and ANXA1 in the skin of patients with CL are shown in Table 3. IFN-γ and TNF-α levels were elevated at the skin of patients with ECR lesions cured within 90 days (P<0.05) and higher in patients with EGR lesions (P<0.05) cured within 180 days. In ENR patients, only TNF-α levels were higher (P<0.05) in patients cured in 90 days. IL-6 levels did not differ between groups. IL-9 and IL-10 levels were higher (P<0.05) in patients with ECR and EGR lesions who were cured after 180 days. Cytokines TGF-β, IL-21, and IL-23 showed an increase in patients with ECR and EGR lesions (P<0.05) who were cured after 180 days and did not differ among patients with ENR lesions. Finally, ANXA1 cytoplasmic expression in M1 macrophages (Figure 2A-C) showed no differences between patients and lesion types. M2 macrophages (Figure 2D-F), but showed higher levels of ANXA1 in ECR skin lesions (P<0.05) of patients cured after 180 days.

**TABLE 3:** Analysis of ANXA1 expression in M1 and M2 macrophages in histological lesions with cutaneous leishmaniasis.

Type of injury	Cure time	IFN-γ	TNF-α	IL-1β	IL-6	IL-9	IL-10	TGF-β	IL-17	IL-21	IL-23	ANXA1 M1	ANXA1 M2
ECR	cure up to 90 days	645.2 ± 357.5 *	496.7 ± 168.6 *	362.4 ± 345.6	199.3 ± 53.7	3.8 ± 1.6	6.1 ± 3.2	45.7 ± 21.3	2.2 ± 1.3	5.2 ± 1.4	5.2 ± 1.4	95.0 ± 23.4	112.3 ± 2.5
	cure up to 180 days	307.4 ± 153.9	286.2 ± 101.2	243.0 ± 174.4	282.8 ± 143.8	12.7 ± 6.2 **	15.7 ± 8.2 **	76.7 ± 29.3 *	2.8 ± 1.2	18.7 ± 6.3 ***	18.7 ± 6.3 ***	111.5 ± 16.3	163.0 ± 9.9 **
EGR	cure up to 90 days	322.2 ± 90.8	305.5 ± 20.5	259.8 ± 172.5	120.5 ± 13.4	4.5 ± 2.1	13.1 ± 1.4	125.2 ± 7.4	2.5 ± 0.7	11.2 ± 1.2	11.2 ± 1.3	117.5 ± 24.8	125.5 ± 6.4
	cure up to 180 days	616.3 ± 132.5 *	517.5 ± 72.8 *	510.8 ± 331.9	119.0 ± 26.9	13.0 ± 4.2 *	23.0 ± 4.2 *	156.8 ± 4.5 *	5.5 ± 2.1	15.7 ± 2.4 *	16.2 ± 1.7 *	118.0 ± 2.8	132.5 ± 10.6
ENR	cure up to 90 days	533.5 ± 103.9	623.0 ± 31.2	147.5 ± 17.7	121.0 ± 26.9	13.0 ± 4.2	16.0 ± 1.4	67.7 ± 13.3	6.0 ± 2.8	23.1 ± 4.4	23.1 ± 4.4	119.0 ± 15.6	143.0 ± 32.5
	cure up to 180 days	605.5 ± 252.4	484.0 ± 113.9 *	148.0 ± 30.4	171.0 ± 87.7	19.5 ± 6.4	21.5 ± 3.5	67.0 ± 2.9	5.5 ± 2.1	22.7 ± 2.5	22.7 ± 2.5	118.0 ± 14.1	150.0 ± 8.5

Data was mean ± SD and was compared by One Way ANOVA, with Tukey post-test. * P<0.05 for 90 days versus 180 days; ** P<0.01 for 90 days versus 180 days; *** P<0.001 for 90 days versus 180 days.

## DISCUSSION

The clinical outcomes and therapeutic responses of patients with CL depend on various factors, including the immunological phenotype, the presence of macrophages at the lesion, and the release of molecular mediators that regulate pro-inflammatory and pro-resolution pathways. In this study, we described the histopathological process at the lesion site, the presence of cytokines, and ANXA1 during infection with *L. braziliensis*, and analyzed these events with the clinical outcome of lesion resolution.

The data presented in this study describe samples from patients with *L. braziliensis* infection exhibiting the typical clinical manifestations of CL, predominantly located in the lower limbs. Most of the patients were male, with diverse skin colors. Similar results have been previously described for this region^16^ ^,^ ^17^ ^,^ ^21^.

Notably, most patients had ECR lesions, indicating early disease detection. Similar findings have been described previously^16^ ^,^ ^17^ ^,^ ^21^. These patients had uncomplicated CL and were treated with meglumine antimoniate, a drug used for CL treatment^1^ ^,^ ^22^ ^,^ ^23^. The efficacy of this treatment, in which *L. braziliensis* predominated, varied from 51.1% to 90%. In Brazil, the therapeutic efficacy of this drug ranges from 46% to 75%^1^ ^,^ ^3^ ^,^ ^23^, allowing its use in uncomplicated cases of CL.

Among patients with ECR lesions that healed within 90 days, there werewas a higher number of M1 macrophages, whereas patients with ECR and EGR lesions that resolved within 180 days had an increased number of M2 macrophages. The cellular population that supports *Leishmania* persistence/proliferation is not well-defined and appears to vary between infections with each parasite species^2^ ^,^ ^5^ ^,^ ^8^. In *L. major*-infected murine models, M2 macrophages were linked to the anti-inflammatory cytokines (e.g., IL-4, IL-10, IL-13, TGF-β), macrophage colony-stimulating factor, arginase I expression (reducing nitric oxide production), parasite survival, and disease progression^8^ ^,^ ^13^ ^-^ ^15^. In contrast, M1 macrophages were associated with the release of inflammatory cytokines (TNF-α, IL-1β, IL-12, IFN-γ), ROS and nitric oxide, culminating in parasite elimination^10^ ^-^ ^12^. However, in *L. braziliensis* infections, it is unclear which host cells primarily supports parasite persistence/proliferation^2^ ^,^ ^8^. Some studies have suggested that M2-like cells, characterized by high arginase activity and low NO production, may be important in promoting more severe diseases^13^. Despite this, it is important to note that M2 macrophages play a crucial role in wound healing in CL, involving numerous growth factors and chemokines^13^ ^-^ ^15^ ^,^ ^26^. In contrast, a robust M1 response may be related to the severe manifestations associated with inflammation induced by *L. braziliensis* ^24^ ^,^ ^25^.

In this study, patients with ECR lesions who achieved healing within 90 days exhibited high levels of IFN-γ and TNF-α, along with an increased degree of M1 macrophages and lower ANXA1 expression o. In contrast, patients with ECR lesions who achieved healing in 180 days exhibited higher levels of TGF-β, IL-9, IL-10, IL-21, and IL-23, alongside a higher number of M2 macrophages and enhanced expression of ANXA1. Patients with EGR lesions, achieving resolution at 180 days, demonstrated increased levels of IFN-γ, TNF-α, TGF-β, IL-9, IL-10, IL-21, and IL-23, an increased number of M2 macrophages, and increased ANXA1 expression. Additionally, patients with ENR lesions and healing in 90 days exhibited an increase in TNF-α levels. These data suggest a complex interplay between specific cytokines and macrophage phenotypes, which are closely related to the infectious microenvironment and clinical outcomes in patients with *L. braziliensis*-induced infection. Some studies have described cytokine levels in human skin during *L. braziliensis* infections^7^ ^,^ ^8^ ^,^ ^15^. These data are of interest for evaluation, particularly when considering an infectious microenvironment. In patients with ECR lesions, these cells are diffusely distributed in the dermis. For EGR lesions, cells are contained in an in situ system that maintains subpopulations of M1 and M2 macrophages, as described in other research^27^. A granuloma creates a condition in which cells are kept in a continuous stimulus system (feedback loops), generating substantial heterogeneity in the types of infected host cells and affecting the parasite replicative state^27^ ^,^ ^28^. In ENR lesions, in addition to the infectious process of the parasite, necrosis exacerbates the immune response, affecting the cellular balance^20^.

Considering the M1/M2 paradigm, M1 activation induces *Leishmania* elimination, whereas M2 is associated with wound healing and potentially exacerbates the disease^8^ ^,^ ^11^ ^,^ ^15^, some molecules have been described to regulate these macrophages. One such molecule is ANXA1, an immunomodulatory protein involved in inflammatory response and macrophage activation^18^ ^,^ ^19^. ANXA1 has been described as a mediator of macrophage opsonization and non-phlogistic phagocytosis^19^ ^,^ ^29^. Additionally, ANXA1 can modulate important cytokines (e.g, TNF-α, IL-1β, and IL-10)^18^, potentially influencing differentiation towards M2 macrophages. Several studies have reported the presence of ANXA1 in the CL^16^ ^,^ ^17^ ^,^ ^21^. One of these works related to ANXA1 action in the phagocytic activity of neutrophils infected with *L. braziliensis* ^21^. Another study identified the differentiation of ANXA1 expression in macrophages subtypes^16^. This study further elucidated ANXA1 in M1 and M2 cells, where its expression in M2 macrophages was higher in patients who healed after 180 days.

A limitation of this study is that our findings do not provide a longitudinal follow-up to assess the durability of healing and potential recurrence of lesions, which is crucial for understanding the long-term efficacy of treatments. The number of patients was limited and may not fully represent the population affected by cutaneous leishmaniasis. In addition, the study did not evaluate the effect of different treatment regimens or adherence levels on the immunological profiles and healing outcomes of CL.

In conclusion, this study suggests that the *L. braziliensis*-induced infectious microenvironment affects the differentiation of M1 and M2 macrophages, cytokine levels, and ANXA1 expression, thereby altering the healing capacity of patients. Therefore, histopathological and immunological investigations may improve the selection of CL therapy.

## References

[1] Brasil. Ministério da Saúde. Secretaria de Vigilância em Saúde.
Departamento de Vigilância das Doenças Transmissíveis (2017). Manual de vigilância da leishmaniose tegumentar.

[2] Caridha D, Vesely B, van Bocxlaer K, Arana B, Mowbray CE, Rafati S (2019). Route map for the discovery and pre-clinical development of new
drugs and treatments for cutaneous leishmaniasis. Int J Parasitol Drugs Drug Resist.

[3] World Health Organization (WHO) (2022). Operational manual on leishmaniasis vector control, surveillance,
monitoring and evaluation.

[4] Brasil. Ministério da Saúde. Secretaria de Vigilância em Saúde.
Departamento de Vigilância das Doenças Transmissíveis (2024). Casos de leishmaniose tegumentar. Brasil, Grandes Regiões e Unidades
Federadas. 2000 a 2022.

[5] Gollob KJ, Viana AG, Dutra WO (2014). Immunoregulation in human American leishmaniasis: balancing
pathology and protection. Parasite Immunol.

[6] Martinez FO, Gordon S, Locati M, Mantovani A (2006). Transcriptional profiling of the human monocyte-to-macrophage
differentiation and polarization: new molecules and patterns of gene
expression. J Immunol.

[7] Sandoval Pacheco CM, Araujo Flores GV, Gonzalez K, de Castro Gomes CM, Passero LFD, Tomokane TY (2021). Macrophage Polarization in the Skin Lesion Caused by Neotropical
Species of Leishmania sp. J Immunol Res.

[8] Bogdan C (2020). Macrophages as host, effector and immunoregulatory cells in
leishmaniasis: Impact of tissue micro-environment and
metabolism. Cytokine X.

[9] Lawrence T, Natoli G (2011). Transcriptional regulation of macrophage polarization: enabling
diversity with identity. Nat Rev Immunol.

[10] Carneiro MB, Lopes ME, Hohman LS, Romano A, David BA, Kratofil R (2020). Th1-Th2 Cross-Regulation Controls Early Leishmania Infection in
the Skin by Modulating the Size of the Permissive Monocytic Host Cell
Reservoir. Cell Host Microbe.

[11] Carneiro MB, Vaz LG, Afonso LCC, Horta MF, Vieira LQ (2021). Regulation of macrophage subsets and cytokine production in
leishmaniasis. Cytokine.

[12] Díaz-Gandarilla JA, Osorio-Trujillo C, Hernández-Ramírez VI, Talamás-Rohana P (2013). PPAR activation induces M1 macrophage polarization via
cPLA₂-COX-2 inhibition, activating ROS production against Leishmania
mexicana. Biomed Res Int.

[13] Krzyszczyk P, Schloss R, Palmer A, Berthiaume F (2018). The Role of Macrophages in Acute and Chronic Wound Healing and
Interventions to Promote Pro-wound Healing Phenotypes. Front Physiol.

[14] Teixeira MV, Soares SAE, Souza VA, de Souza Marques AM, de Almeida Soares CM, Baeza LC (2022). Murine macrophages do not support the proliferation of Leishmania
(Viannia) braziliensis amastigotes even in absence of nitric oxide and
presence of high arginase activity. Parasitol Res.

[15] Bogdan C (2020). Macrophages as host, effector and immunoregulatory cells in
leishmaniasis: Impact of tissue micro-environment and
metabolism. Cytokine X.

[16] Silva JMD, Silva HALD, Zelenski C, Souza JAM, Hueb M, Damazo AS (2019). Analysis of macrophage subtypes and annexin A1 expression in
lesions of patients with cutaneous leishmaniasis. Rev Soc Bras Med Trop.

[17] Silva HA, Lima GS, Boité MC, Porrozzi R, Hueb M, Damazo AS (2015). Expression of annexin A1 in Leishmania-infected skin and its
correlation with histopathological features. Rev Soc Bras Med Trop.

[18] Damazo AS, Yona S, D'Acquisto F, Flower RJ, Oliani SM, Perretti M (2005). Critical protective role for annexin 1 gene expression in the
endotoxemic murine microcirculation. Am J Pathol.

[19] Sugimoto MA, Vago JP, Teixeira MM, Sousa LP (2016). Annexin A1 and the Resolution of Inflammation: Modulation of
Neutrophil Recruitment, Apoptosis, and Clearance. J Immunol Res.

[20] de Magalhães AV, Moraes MA, Raick AN, Llanos-Cuentas A, Costa JM, Cuba CC (1986). Histopatologia da leishmaniose tegumentar por Leishmania
braziliensis braziliensis. 4. Classificação histopatológica
(1). Rev Inst Med Trop Sao Paulo.

[21] Pona MN, Dietrich JM, Silva JMD, Silva HALD, Hueb M, Damazo AS (2021). Analysis of annexin-A1 in the macrophages and apoptotic cells of
patients with cutaneous leishmaniasis. Rev Soc Bras Med Trop.

[22] Kocyigit A, Gur S, Gurel MS, Bulut V, Ulukanligil M (2002). Antimonial therapy induces circulating proinflammatory cytokines
in patients with cutaneous leishmaniasis. Infect Immun.

[23] Deps PD, Viana MC, Falqueto A, Dietze R (2000). Avaliaçao comparativa da eficácia e toxicidade do antimoniato de
N-metil-glucamina e do estibogluconato de sódio BP88 no tratamento da
leishmaniose cutânea localizada. Rev Soc Bras Med Trop.

[24] Gaze ST, Dutra WO, Lessa M, Lessa H, Guimarães LH, Jesus AR (2006). Mucosal leishmaniasis patients display an activated inflammatory
T-cell phenotype associated with a nonbalanced monocyte
population. Scand J Immunol.

[25] Giudice A, Vendrame C, Bezerra C, Carvalho LP, Delavechia T, Carvalho EM (2012). Macrophages participate in host protection and the disease
pathology associated with Leishmania braziliensis infection. BMC Infect Dis.

[26] Lee SH, Charmoy M, Romano A, Paun A, Chaves MM, Cope FO (2018). Mannose receptor high, M2 dermal macrophages mediate nonhealing
Leishmania major infection in a Th1 immune environment. J Exp Med.

[27] Kaye PM, Beattie L (2016). Lessons from other diseases: granulomatous inflammation in
leishmaniasis. Semin Immunopathol.

[28] Saunders EC, McConville MJ (2020). Immunometabolism of Leishmania granulomas. Immunol Cell Biol.

[29] Yona S, Heinsbroek SE, Peiser L, Gordon S, Perretti M, Flower RJ (2006). Impaired phagocytic mechanism in annexin 1 null
macrophages. Br J Pharmacol.

